# Implicit Affective Rivalry: A Behavioral and fMRI Study Combining Olfactory and Auditory Stimulation

**DOI:** 10.3389/fnbeh.2018.00313

**Published:** 2018-12-18

**Authors:** Mark Berthold-Losleben, Ute Habel, Anne-Kathrin Brehl, Jessica Freiherr, Katrin Losleben, Frank Schneider, Katrin Amunts, Nils Kohn

**Affiliations:** ^1^Division of Mental Healthcare, St. Olavs University Hospital, Trondheim, Norway; ^2^Norwegian University of Science and Technology (NTNU), Trondheim, Norway; ^3^Uniklinik RWTH Aachen, Aachen, Germany; ^4^Donders Institute for Brain, Cognition and Behaviour, Radboud University, Nijmegen, Netherlands; ^5^Fraunhofer Institute for Process Engineering and Packaging (IVV), Freising, Germany; ^6^UiT The Arctic University of Norway, Tromsø, Norway; ^7^Düsseldorf University Hospital, Düsseldorf, Germany; ^8^Institute of Neuroscience and Medicine, Jülich Research Centre, Jülich, Germany

**Keywords:** music, olfaction, fMRI, emotion regulation, gender

## Abstract

Aversive odors are highly salient stimuli that serve a protective function. Thus, emotional reactions elicited by negative odors may be hardly influenceable. We aim to elucidate if negative mood induced by negative odors can be modulated automatically by positively valenced stimuli. We included 32 healthy participants (16 men) in an fMRI design combining aversive and neutral olfactory stimuli with positive and neutral auditory stimuli to test the influence of aversive olfactory stimuli on subjective emotional state and brain activation when combined with positive and neutral auditory stimuli. The behavioral results show an interaction of negative olfactory stimuli on ratings of disgust, perceived valence of music, and subjective affective state, while positive auditory stimulation did not show this interaction. On a neuronal level, we observed main effects for auditory and olfactory stimulation, which are largely congruent with previous literature. However, the pairing of both stimuli was associated with attenuated brain activity in a set of brain areas (supplementary motor area, temporal pole, superior frontal gyrus) which overlaps with multisensory processing areas and pave the way for automatic emotion regulation. Our behavioral results and the integrated neural patterns provide evidence of predominance of olfaction in processing of affective rivalry from multiple sensory modalities.

## Introduction

Emotions and their regulation affect our social interaction, our well-being and influence what kind of decisions we make, therefore playing an important role in our everyday life.

Emotions have been described as a perception-valuation-action sequence (Etkin et al., 2015), in which an affective stimulus is perceived, internal evaluation is made and this leads to an action, either physically or mentally. This may occur with or without conscious awareness (Lazarus, 1991). Interestingly, the regulation of emotion shares a perception valuation action sequence with emotional processing, despite being conceptually different (Etkin et al., 2015). Emotion regulation has long been discussed as a cognitive, deliberate process. Nevertheless, automatic processes that are not intentionally initiated or guided also seem to play an important role in the broader picture of emotion reactivity and regulation (Gross, 1998; Mauss et al., 2006, 2007; Kohn et al., 2011; Etkin et al., 2015). This differentiation is supported by findings of studies showing different brain regions involved in either consciously controlled or automatic, non-intentional emotional regulatory processes (Ochsner et al., 2009; Gyurak et al., 2011). The ventral ACC and the ventromedial PFC have been suggested as neural correlates of automatic emotion regulation (Etkin et al., 2015). Interestingly, areas widely believed to be solely important for motor functions like the cerebellum (Schutter and Van Honk, 2009; Strata et al., 2011; Baumann and Mattingley, 2012; Stoodley et al., 2012) or Brodmann Area 6, known as premotor cortex and supplementary motor area, also seem to be involved in cognitive and automatic up- and down-regulation of negative emotions (Ochsner et al., 2004; Kohn et al., 2011, 2014, 2015; Buhle et al., 2013; Pawliczek et al., 2013; Morawetz et al., 2017).

Inducing emotions by external stimuli is a well-established method and has been successfully utilized in many studies (for reviews see: Gerrards-Hesse et al., 1994; Westermann et al., 1996). Among the negatively valenced (basic) emotions, disgust is probably the emotion relying most strongly on external stimuli (for a discussion of disgust see: Rozin et al., 2008). Disgust can be induced by several methods, such as presentation of faces showing disgust, disgusting pictures, or with gustatory or olfactory stimuli. Our olfactory system is strongly associated with processing disgust (Croy et al., 2011); it may, from an evolutionary point of view, even be the most important feature in olfaction (Stevenson, 2010), and thus is a highly relevant target for the investigation of interactive modulation properties as it may play an important role in automatic emotion regulation (Mauss et al., 2007). Aversive odors like rotten yeast, hydrogen sulfide (rotten eggs), or isovaleric acid (dirty socks odor) are appropriate stimuli for experimental induction of disgust and have been successfully used to induce disgust as a major negative emotion (Habel et al., 2007a; Seubert et al., 2009, 2010a). Importantly, behavioral studies suggest that the underlying affective processing when using negative olfactory stimuli is presumably rather an automatic than a conscious mechanism, since negative odors are detected and evaluated faster than positive or neutral odors (Bensafi et al., 2003). An initial negative affective reaction to aversive olfactory stimuli is probably hardly influenceable, which makes sense, considering the fact, that aversive olfactory stimuli are evolutionary important due to their protective functions (Ache and Young, 2005; Rozin et al., 2008). Also anatomically, odor induced emotions in general, and in our case negative emotions in particular, are initially “hard-wired”: The olfactory system combines phylogenetically old and new brain areas, is unique in its predominant access of ipsilateral regions, and is considered to be the only sensory system with direct connections (not entailing a thalamic relay) to the ipsilateral, superficial parts of the amygdala (Amunts et al., 2005; Lundström et al., 2011; Bzdok et al., 2013; Seubert et al., 2013a). In general, fMRI and PET studies revealed the amygdala, the orbitofrontal cortex (OFC), the insula, the superior temporal gyrus, the anterior cingulate gyrus, the piriform cortex, and the hypothalamus (Zald and Pardo, 1997; Lundström et al., 2011; Seubert et al., 2013a,b) to be involved in processing olfaction. Moreover, especially anterior parts of the insula were found to be associated with disgust in several former studies, induced either by visual stimuli (Phillips et al., 1997; Sprengelmeyer et al., 1998; Wicker et al., 2003; von dem Hagen et al., 2009), in paradigms using odors (Reske et al., 2010; Lundström et al., 2011), and in a study using faces for inducing disgust, primed by negatively valenced olfactory stimuli (Seubert et al., 2010b). Olfactory stimulation is therefore a promising tool for the automatic induction of a negative affective state.

Yet, in real life circumstances, we are rarely confronted with just one single emotional stimulus in just one modality. In order to investigate multi-modal automatic emotion generation and regulation, we aimed to use another stimulus modality, which also induces emotions automatically (Dyck et al., 2011), and combine an opposing emotional quality with disgusting, automatic olfaction. Listening to classical music is a strong stimulus for inducing different emotions like happiness, sadness, or even neutral emotional states (Mitterschiffthaler et al., 2007; Koelsch, 2010, 2014). We understand music as involving automatic as well as cognitive pathways due to its complex nature (Dyck et al., 2011). Or to follow Brattico and Jacobsen (2009), one can either unconsciously like (or dislike) or consciously judge music.

Mitterschiffthaler et al. (2007) found significant BOLD responses associated with happy relative to neutral music in the left superior and medial frontal gyrus, left ACC, left posterior cingulate, bilateral primary auditory cortex, bilateral ventral striatum, left caudate nucleus, left parahippocampal gyrus, and left precuneus. Other regions involved in processing pleasant contrasted to unpleasant music were the left anterior superior insula, the IFG, and rolandic and frontal opercular areas (Koelsch et al., 2006; Koelsch, 2014).

In combining negative olfactory and positive auditory stimuli we created a scenario, where two conflicting stimuli that both generate emotional reactivity in an automatic fashion have to be integrated into one percept. It has been studied in detail how unitary perceptual experiences are created through multisensory integration (Stein and Stanford, 2008). These studies mainly focused on integration of auditory, visual, and tactile stimuli (Driver and Noesselt, 2008). To avoid semantic inconsistencies we define the emotional response as integrated percept (Stein et al., 2010) derived from the administration of cross-modal, emotion inducing stimuli. Furthermore, we investigate a multisensory process rather than multisensory integration due to the complexity of our integrated percept.

This behavioral and fMRI study was designed as part of a larger project that aimed to investigate if a structured low-level music listening training would be able to influence automatic emotion generation and regulation of automatic olfactory emotional reactivity. In this manuscript, we present the results from the first pre-training measurement.

Besides providing the baseline for the subsequent training, this measurement was set up to validate the paradigm: a negative olfactory stimulus and a positive auditory stimulus are independently capable of inducing a congruent emotional state and elicits reliable BOLD responses to both music and olfaction in respective primary and secondary sensory areas. Based on the anatomical connectivity of the olfactory sensory system and its evolutionary salience, for the pairing of a negative olfactory with a positive auditory stimulus, we hypothesized the olfactory stimulus dominates the emotional self-rating. Furthermore, we expect interactions of music and olfaction in multisensory processing and related attentional brain areas (Mauss et al., 2007; Talsma et al., 2010; Kohn et al., 2011; Talsma, 2015). Based on previous results we expect activation in multisensory processing areas, such as premotor cortex, superior temporal, and parietal gyrus (Driver and Noesselt, 2008; Talsma, 2015; Hartcher-O'Brien et al., 2017). Evaluation of disgust potentially moderates brain activity in somatosensory regions (Croy et al., 2016). As multisensory integration and processing serves automatic emotion regulation, we furthermore expect to observe brain activation in areas related to emotion generation and automatic emotion regulation such as the insula, amygdala, and the orbitofrontal cortex (Gyurak et al., 2011; Etkin et al., 2015). The latter has also been implicated in multisensory integration (Thesen et al., 2004). As gender has been shown to moderate emotion processing (Cahill, 2006; Kohn et al., 2011), we included gender as a factor in our analyses of behavioral results.

In every-day life we are often confronted with combinations of multimodal and incongruent stimuli, be it by chance or systematically with an intention to cause a specific emotional state or reaction (e.g., to elicit a desire in a customer), yet this phenomenon has, as far as we know, rarely been studied.

## Materials and Methods

### Subjects

Sixteen female and 16 male volunteers aged 18–32 years (mean age = 25.00 years, SD = 3.30) participated in the experiment. The mean educational level was 13.00 years of school (SD = 0.00), all participants were qualified for university education admission. The participants did not habitually listen to music more than 60 min per day and did not play or practice any instrument in the last 12 months. No subject had acute or chronic sinusitis or reported diminished olfactory sensitivity.

All participants were right-handed and had no history of neurological or psychiatric disorders or severe head trauma and no known abnormalities regarding olfactory and auditory function, which was tested by the use of the semi-structured interview SCIDPIT (Demal, 1999).

Subjects gave written and verbal informed consent prior to participation in the study. The study was approved by the ethics committee of the Medical School, RWTH Aachen University, Germany and was carried out in accordance with the Declaration of Helsinki (Williams, 2008).

### Auditory Stimuli

#### Pre-evaluation Study of the Auditory Stimuli

The positive auditory stimuli were extracted from classical music pieces, successfully used in previous studies to induce positive emotional states (Halberstadt et al., 1995; Peretz and Hébert, 2000; Mitterschiffthaler et al., 2007; Koelsch, 2010, 2014; van Tricht et al., 2010). In the cutting process, harmonic aspects of the sequence were taken carefully into account. The collection consisted of 45 sequences taken from 18 different pieces and 11 different composers and additionally 16 different musical scales played polyphonically on a piano, each with a duration of 16 s. Thirty-two healthy volunteers (mean age = 30.25 years, SD = 6.64; gender ratio = 18 women; 14 men), who did not take part in the main study, participated in a preceding pilot study. Their task was to evaluate the valence of these 61 sequences on an interval scale from −4 (= very negative) over 0 (= neutral) to +4 (= very positive). The stimuli and rating scales were presented via a computer with headphones using the software Presentation (Neurobehavioral Systems Inc., Berkeley CA, USA). The responses of the subjects entered using a standard keyboard and logged in Presentation.

#### Auditory Stimuli For the fMRI Task

Of the 11 sequences rated most positively (≥2.00) in the preceding pilot study, we took 8 sequences into the fMRI experiment, which differed most widely regarding the pieces and composers. The neutral stimuli consisted of the 4 sequences rated most neutrally. Among these were 2 musical scales and 2 sequences of musical pieces, providing a better comparability to the positive auditory stimuli regarding the composition complexity (e.g., rhythm, timbre, tempo). For an overview of the positive and neutral auditory stimuli taken for the fMRI task, see Table 1. The volume of the sequences was leveled and adjusted to each participant individually before the experiment, to assure that the sound was clearly perceivable.

**Table 1:** Pre-evaluation of auditory stimuli for the fMRI task.

**Composer**	**Piece**	**Part**	***n*** **= 32**		**Category**
			**Mean rating**	**SD**	
Bizet	Carmen	Chanson du Toréador	2.813	0.222	positive
Bizet	Carmen	Chanson du Toréador	2.750	0.266	positive
Mozart	Serenade no. 13	Rondo	2.719	0.192	positive
Prokofjew	Peter and the Wolf	Peter in the Meadow	2.625	0.205	positive
Mozart	Serenade no. 13	Allegro	2.531	0.258	positive
Mozart	Divertimento in D	Presto	2.344	0.244	positive
Saint-Saëns	Carnival of the Animals	Finale	2.219	0.194	positive
Vivaldi	Concerto for 2 mandolines and strings	Allegro	2.188	0.244	positive
	Scale	C major down	0.000	0.269	neutral
Mozart	Clarinet concerto	Adagio	0.000	0.321	neutral
	Scale	C major up	−0.063	0.308	neutral
Beethoven	Piano concert no. 4	Allegro moderato	−0.094	0.357	neutral

*Mean rating on interval scale from −4 (very negative) to +4 (very positive) with standard deviation (SD) of 32 participants. Pre-evaluation was performed with 45 classical music sequences and 16 polyphonical music scales (all cut with a duration of 16 s taken harmonic aspects into account). Category shows if taken as positive or neutral stimulus for fMRI task*.

### Olfactory Stimuli

The participants were exposed to an olfactory condition [the unpleasant odor H_2_S (hydrosulphide) in nitrogen] and a neutral baseline condition, during which the regular ambient airflow was held constant with no odor superimposed. Both stimuli were delivered through a tube terminating in a nose piece that was inserted into the nostril about 1 cm deep. The application was unirhinal on the right side, which shows reliable results regarding the BOLD responses as described in several studies (Zatorre and Jones-Gotman, 1990; Broman et al., 2001; Seubert et al., 2010a). For the stimulus presentation a Burghart OM4 olfactometer (Burghart Medizintechnik, Wedel, Germany) was used, which operates at a constant temperature of 40°C to reduce thermal irritation of the nasal mucosa. The olfactometer delivered a constant humidified airflow for both conditions, set to 7.0 l/min. Thus, the odor or ambient air reaches the olfactory epithelium without mucosal dehydration and without the participant having to sniff. This minimizes inter-individual effects of breathing habits. During the stimulus presentation the participants were asked to respire orally only. A velopharyngeal closure was not required because of the very intense odor presentation. H_2_S was presented at a constant flow of 4.3 l/min diluted with 2.7 l/min ambient air resulting in a 12.3 ppm concentration of the H_2_S stimuli. H_2_S presented in this manner, has been shown to successfully induce the negative emotional state of disgust (Seubert et al., 2009) without trigeminal impact (Doty et al., 1978).

### fMRI Task And Valence Ratings

**Figure 1:**
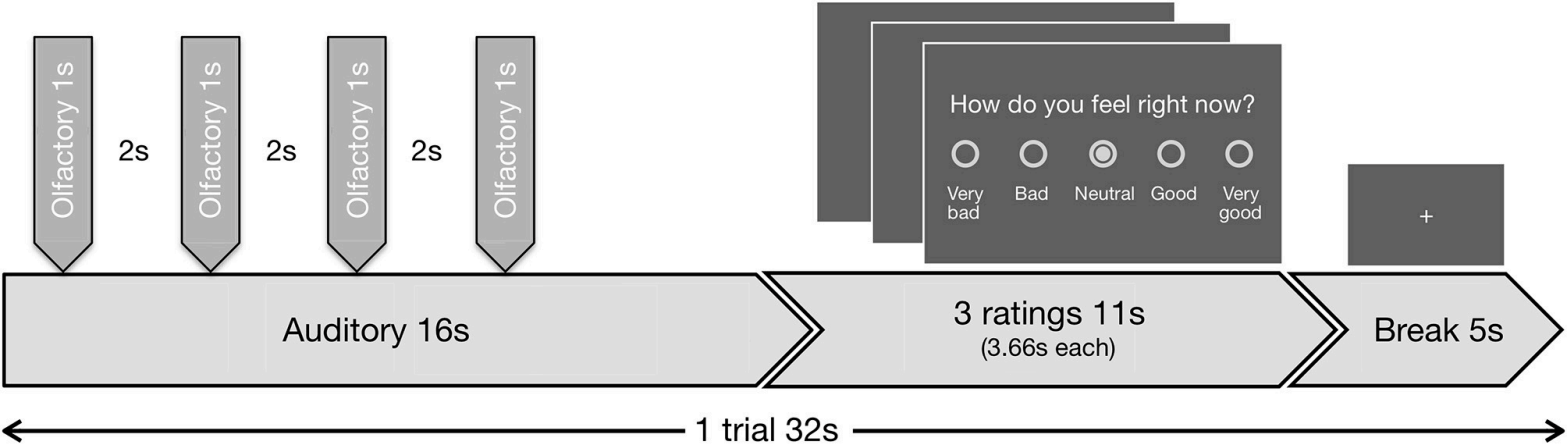
Schematic visualization of the experimental design. Exemplary trial (duration 32 s) out of 48 trials, presented in the fMRI task. Each trial began with the presentation of an auditory stimulus (duration 16 s, first second fade-in, last second fade-out). Four olfactory stimuli (duration 1 s each, interrupted by breaks of 2 s) were administered during the continuing presentation of the auditory stimulus. All four of the olfactory stimuli were either negative or neutral. The first olfactory pulse was administered jittered at random with a delay of 1.0, 1.5, 2.0, or 2.5 s relative to the beginning of the auditory stimulus, so always after the fade-in period was completed. The administration of the stimuli combination was followed by three ratings (duration 11 s, 3.66 s per rating) and a baseline period (duration 5 s), where a fixation cross was shown.

The task included 48 blocks. During each block, in the first 16 s the auditory in combination with the olfactory stimuli were presented, followed by three valence ratings with a total duration of 11 s and finally a baseline period (fixation cross) of 5 s (Figure 1). During the valence ratings, the subjects had to indicate on a 5-point scale (a) how disgusting they would rate the smell (0 = not at all to 4 = extremely), (b) how they would rate the music (−2 = very negative to 2 = very positive; 0 = neutral), and (c) how they currently feel (−2 = very bad to 2 = very good; 0 = neutral). During stimulus presentation and baseline period a fixation cross was visible. Disgust and valence of music reflect the lower level emotional perception of the stimuli, while the emotional state reflects higher order regulatory processes.

The auditory stimuli (A) were either neutral (A^0^) or positive (A^+^) and were faded in and out during the first and last seconds, respectively by linear volume progression/regression.

Simultaneously either normal air as neutral (O^0^) or H_2_S as negative (O^−^) olfactory condition (O) was presented. To minimize habituation effects H_2_S was applied in 4 pulses with duration of 1 s each, interrupted by breaks of 2 s in between. Similar approaches have previously been successfully used without showing evidence of habituation effects (Habel et al., 2007b; Koch et al., 2007; Schneider et al., 2007). The beginning of the first olfactory pulse was jittered at random with a delay of 1.0, 1.5, 2.0, or 2.5 s relative to the beginning of each auditory stimulus. We planned to exclude subjects that deviated with more than 2 standard deviations in the disgust ratings to the olfactory condition to rule out abnormal olfactory sensitivity.

The combination of the two different stimuli in each condition resulted in a 2 × 2 experimental design. Each combination (A^0^O^−^, A^0^O^0^, A^+^O^−^, A^+^O^0^) was presented 12 times in total while the order of appearance was randomized.

For the visual presentation in the scanner and the interaction during the ratings the software Presentation (Neurobehavioral Systems Inc., Berkeley, CA, USA) was used. The subjects gave their responses by moving a cursor to the desired location via button boxes positioned comfortably under their right arm (LumiTouch, Photon Control, Burnaby, Canada).

### fMRI Data Acquisition

Functional imaging was performed on a 3T Trio MR Scanner (Siemens Medical Systems, Erlangen, Germany) equipped with a 12-channel head matrix coil and using echo-planar imaging (EPI) sensitive to BOLD contrast (whole brain, T2^*^, voxel size: 3.4 × 3.4 × 3.3 mm^3^, matrix size 64 × 64, field of view [FoV] = 220 mm^2^, 36 axial slices, slice gap = 0.3 mm, acquisition orientation: ascending, echo time [TE] = 30 ms, repetition time [TR] = 2 s, flip angle [α] = 77°, min. 773 volumes (range: 773–800), slice orientation: AC-PC).

### Statistical Data Analysis

#### Behavioral Data

Repeated-measures two-way ANOVAs were calculated for the three dependent variables disgust, music valence and emotional state ratings, respectively using the factors auditory stimulation (A; two levels: A^0^, A^+^) and olfactory stimulation (O; two levels: O^0^, O^+^). Gender was included as between-subject factor. Greenhouse-Geisser corrected *p*-values were used. Significant gender and interaction effects were decomposed by *post-hoc* paired-sample *t*-tests when applicable.

#### fMRI Data Processing

Analyses of functional images were performed with SPM8 (Statistical Parametric Mapping, Welcome Trust Center for Neuroimaging, UCL London, UK). Slice time correction, realignment, stereotaxic normalization, smoothing (8 mm FWHM Gaussian kernel), and high pass-filtering were applied in the given order. The functional scans were realigned with a two-pass procedure: In the first pass, the first scan and in the second pass, the mean scan were used as reference image. The mean EPIs were applied to the unified segmentation approach (Ashburner and Friston, 2005), transformed into standard space (Montreal Neurological Institute templates) and resampled to 2 × 2 × 2 mm^3^. Lastly, images were smoothed using a Gaussian kernel of 8 mm full-width at half-maximum and high-pass filtered (7.81 mHz).

For 1st level analysis four different condition combinations (A^0^O^−^, A^0^O^0^, A^+^O^−^, A^+^O^0^) were defined (block design) in the general linear model framework as implemented in SPM8. Each block was modeled as lasting 16 s (onset to end) and labeled according to the underlying combination of olfactory and auditory stimuli. Additionally, a nuisance regressor was added that included instruction, ratings and other periods of no interest. The onset functions were convolved with the canonical hemodynamic response function (HRF, as implemented in SPM8).

Contrast estimates of the HRF of each condition were taken to the group level. Mirroring the behavioral analyses, a 2 × 2 ANOVA (flexible factorial design) was performed in order to calculate the contrasts of interest. Namely these are: (1) The effect of positive auditory stimulation (contrast positive vs. neutral auditory stimulus presentation; A^+^O^−^ + A^+^O^0^ > A^0^O^−^ + A^0^O^0^, further referred to as A^+^ > A^0^). (2) The effect of negative olfactory stimulation (contrast negative vs. neutral olfactory stimulus presentation; A^0^O^−^ + A^+^O^−^ > A^0^O^0^ + A^+^O^0^, further referred to as O^−^ > O^0^). (3) Interaction of auditory and olfactory stimulation (A^+^O^−^ + A^0^O^0^ vs. A^+^O^0^ + A^0^O^−^, further referred to as A × O). The interaction is implemented in SPM in linear contrasts (Gläscher and Gitelman, 2008). It can either reflect (A) an attenuation of relevant auditory and olfactory brain activity (A^+^O^−^ + A^0^O^0^ < A^+^O^0^ + A^0^O^−^) or (B) an increase of activity in the sense of multimodal integration (elevated olfactory and auditory processing when combined; A^+^O^−^ + A^0^O^0^ > A^+^O^0^ + A^0^O^−^). Each interaction needs further qualification to ensure (for A) that brain areas, that are at all responsive to both olfactory and auditory stimulation, show attenuated activity. Therefore, we calculated a conjunction-null analysis of the interaction and the main effect of positive auditory during neutral olfactory stimulation (A × O) n (A^+^O^0^) and negative olfactory stimulation during neutral auditory stimulation (A × O) n (A^0^O^−^) as well as the three-way conjunction (A × O) n (A^+^O^0^) n (A^0^O^−^). Elevated activity during positive auditory and negative olfactory stimulation (B, multimodal integration) can be qualified by computing the conjunction of the relevant interaction (A^+^O^−^ + A^0^O^0^ > A^+^O^0^ + A^0^O^−^) with activity during positive auditory and negative olfactory stimulation (A × O) n (A^+^O^−^). Additionally, a 2 × 2 × 2 ANOVA including gender as between-subject factor was performed.

In order to correct for multiple comparisons within one volume, the contrasts of the effects of positive music and negative odor as well as the interaction of gender with these effects were FWE-corrected on the voxel level (family-wise error, based on Gaussian random field theory) at *p* = 0.05 with an extent threshold of 5 voxels. For the conjunction-null analysis (Nichols et al., 2005) of the interactions [(A × O) n (A^+^O^0^) n (A^0^O^−^) and (A × O) n (A^+^O^−^)] a significance level at *p* < 0.001 uncorrected for whole-brain volume with an extent threshold of 10 voxels was applied. Given the independence of contrasts in a conjunction, the threshold for whole-brain comparisons can be determined by multiplying the individual thresholds. Thereby a conjunction of two images yields a threshold of *p* < 0.000001, which is below the critical threshold of FWE correction.

For anatomical localization of the functional data we referred to probabilistic cytoarchitectonic maps with the SPM Anatomy Toolbox (Eickhoff et al., 2005).

#### Integration of Behavioral Ratings and Brain Activation

We aimed to investigate whether behavioral ratings show a significant association with brain activation in brain areas that process interaction. For this purpose, we focused on the interaction analyses. From all four significant clusters, we extracted one mean beta values (averaged over all voxels in that cluster) for each cluster. This approach resulted in one beta value per significant cluster and condition. We performed bivariate Pearson's correlations for the brain activation in all four conditions (A^0^O^−^, A^0^O^0^, A^+^O^−^, A^+^O^0^) with the corresponding rating for disgust, music and emotional state

We list correlations with a significance level of *p* < 0.05. Correlations surviving false discovery rate correction (Benjamini and Hochberg, 1995) for 48 *p*-values (*p* < 0.00137 at *q* = 0.05; *p* < 0.013 > 0.00137 at *q* < 0.1 > 0.05) are marked with an asterisk.

## Results

### Behavioral Results

#### Disgust Ratings of Olfactory Stimuli

The effect of the olfactory stimuli (O) on disgust ratings was significant [F_(1, 31)_ = 587.54, *p* < 0.001, $\eta_{\text{p}}^{2}$ = 0.95]. During the negative olfactory conditions, the level of disgust was rated significantly higher compared to the neutral olfactory conditions. There were no significant effects of the auditory stimuli (A) or the interaction A × O on the disgust ratings.

**Figure 2:**
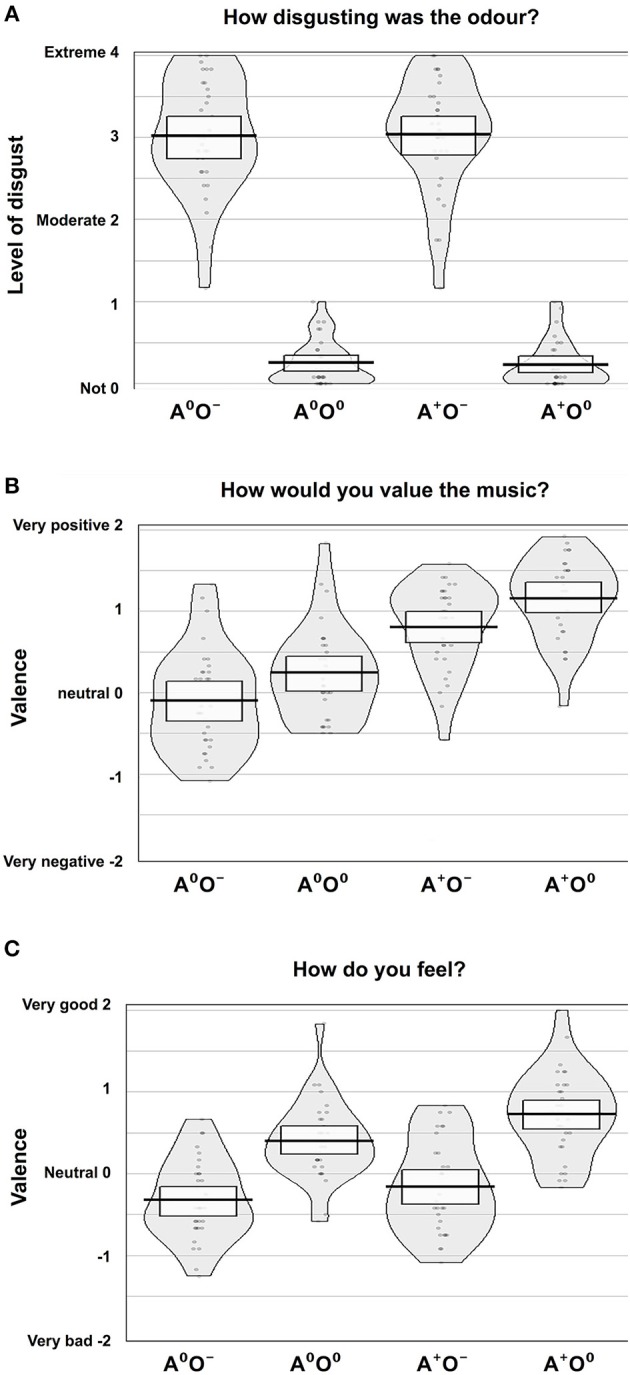
Subjective rating results. Ratings of **(A)** disgust level, **(B)** valence of music, and **(C)** emotional state according to the four conditions, auditory (A; two levels: A^0^, A^+^) and olfactory (O; two levels: O^0^, O^−^).

We were not able to establish a main effect of gender. However, the interaction of the auditory stimuli and gender (A × gender) showed a significant effect on the disgust ratings [F_(1, 30)_ = 4.71, *p* < 0.05; $\eta_{\text{p}}^{2}$ = 0.14]. Male participants showed lower disgust ratings when a positive auditory stimulus was paired with negative olfaction, while in women the combination of positive music and negative odor led to increased report of disgust. Disgust ratings for neutral odor were equally low in both auditory conditions. Although formal significance was not reached (0.05 < *p* < 0.1; no evidence against H0), this pattern may indicate a trend in the three-way interaction of olfaction, audition and gender. Figure 2A shows an overview of the disgust ratings.

#### Valence Ratings of Auditory Stimuli

The effect of the auditory stimuli (A) on the valence ratings of the music was significant [F_(1, 31)_ = 54.68, *p* < 0.001; $\eta_{\text{p}}^{2}$ = 0.64]. There was also a significant effect of the olfactory stimuli (O) on the valence ratings of the music [F_(1, 31)_ = 43.9, *p* < 0.001; $\eta_{\text{p}}^{2}$ = 0.59]. Positive auditory stimuli were rated as significantly more positive compared to the neutral auditory stimuli. Moreover, during neutral compared to negative olfactory stimulation, subjects rated the neutral and positive auditory stimuli more positively. There was no significant effect of the interaction A × O on the valence ratings of the auditory stimuli. Further, gender had no effect. See Figure 2B for an overview of the valence ratings of the auditory stimuli.

#### Ratings of Emotional State

There was a significant effect of the olfactory stimuli on the ratings of the emotional state [F_(1, 31)_ = 99.5, *p* < 0.001; $\eta_{\text{p}}^{2}$ = 0.76]. Under the same auditory condition, the emotional state was rated significantly more positively during neutral compared to negative olfactory stimulation. Also, the auditory stimuli showed a significant effect on the ratings of the emotional state [F_(1, 31)_ = 23.0, *p* < 0.001; $\eta_{\text{p}}^{2}$ = 0.43] i.e., positive auditory stimuli lead to an elevated positive emotional state regardless of olfactory condition. Moreover, there was a significant interaction effect A × O [F_(1, 31)_ = 7.030, *p* < 0.05; $\eta_{\text{p}}^{2}$ = 0.19]. The elevating effect of positive music on emotional state is lower when combined with negative stimuli. Again, gender had no significant effect on ratings of emotional state. Figure 2C shows an overview of the ratings of the emotional states.

### Imaging Results

#### Main Effect of Positive Auditory Stimulation

**Figure 3:**
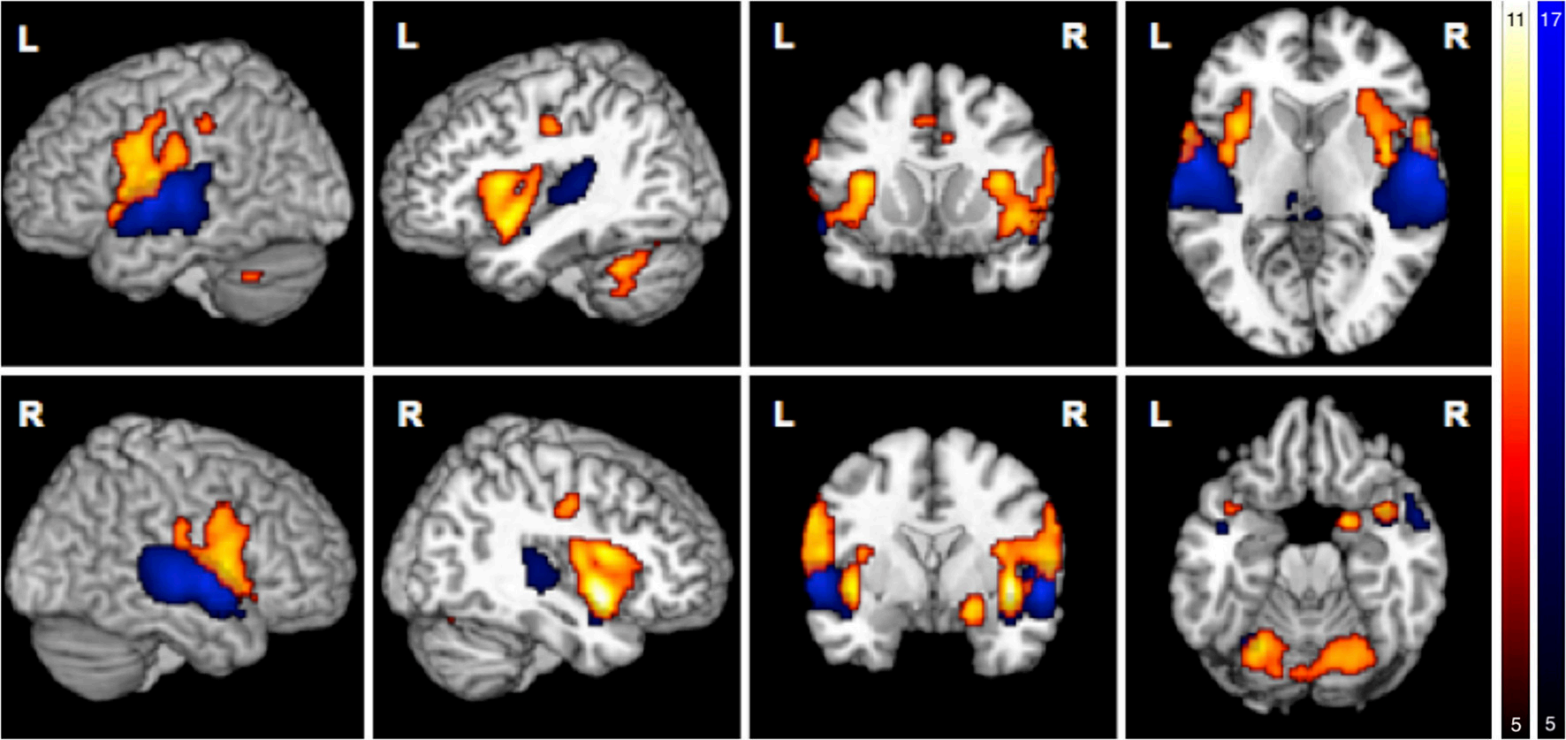
Main effects in brain activity for olfaction and audition. BOLD responses for negative > neutral odor (O^−^ > O^0^) displayed in red. Blue clusters show BOLD responses for positive > neutral music (A^+^ > A^0^). FWE corrected at *p* < 0.05, k > 5.

Simple main effects (condition vs. low level baseline) are reported in Supplementary Tables 1–4. Significant BOLD responses of A^+^ > A^0^ were found bilaterally in the superior temporal lobe involving the primary auditory cortex (TE1.0, TE1.1, TE1.2), and TE3.0, in the right temporal pole, bilaterally in the parietal operculum (OP1, OP4), in the left middle temporal gyrus and inferior parietal cortex (PFcm), bilaterally in the posterior thalamus, bilaterally in granular and dysgranular parts of the posterior insula (lg1, lg2, ld1), and in lobule VI of the left cerebellum (Figure 3/blue, Table 2). No significant BOLD responses of A^0^ > A^+^ were found. Female compared to male participants showed a significantly stronger activation in the right superior temporal lobe (1 cluster with a cluster extent of 37 voxels involving TE1.0, TE3.0, OP4).

**Table 2:** Activations during positive > neutral auditory stimulation (A^+^ > A^0^).

				**MNI coordinates**		
**Area**	**Hemisphere**	**Cluster**	**Cluster extent**	**x**	**y**	**z**	***t*-value**
Superior temporal gyrus	R	C1	3,392			
Primary auditory cortex (TE1.0)				56	−8	−4	17.73
Primary auditory cortex (TE1.1)				40	−28	4	6.62
Primary auditory cortex (TE1.2)				56	−2	−8	16.81
TE3.0				66	−20	−4	13.99
Temporal pole				40	4	−22	5.85
Parietal operculum (OP1, OP4)	R	C1		without peak voxel in the cluster			
Posterior insula (lg1, lg2, ld1)	R	C1		without peak voxel in the cluster			
Superior temporal gyrus	L	C2	3,082
Primary auditory cortex (TE1.0)				without peak voxel in the cluster			
Primary auditory cortex (TE1.1)				−38	−32	8	7.50
Primary auditory cortex (TE1.2)				−56	−6	−2	16.74
TE3.0				−68	−20	0	13.55
Middle temporal gyrus	L	C2		−44	−4	−18	6.10
Parietal operculum (OP1, OP4)	L	C2		without peak voxel in the cluster			
Inferior parietal cortex (PFcm)	L	C2		without peak voxel in the cluster			
Posterior insula (lg1, lg2, ld1)	L	C2		without peak voxel in the cluster			
Posterior Thalamus	L	C3	113	−8	−28	−4	6.30
Posterior Thalamus	R	C4	57	12	−26	−8	5.51
Cerebellum (lobule VI)	L	C5	54	−30	−60	−26	6.27

*FWE-corrected at p < 0.05 with extend threshold of 5 voxels. Presentation of areas with at least 5 % activation and peak voxel if possible*.

#### Main Effect of Negative Olfactory Stimulation

BOLD responses associated with O^−^ > O^0^ were found bilaterally in the anterior insula, bilaterally in the parietal operculum (OP4, OP1), in the right Rolandic operculum (BA44), bilaterally in the temporal pole, bilaterally in the inferior parietal cortex (right: PFop; left: PFop, PFt), bilaterally in the precentral gyrus (right: BA6, BA4a; left: BA6), in the left postcentral gyrus (BA1, BA4p), bilaterally in the primary somatosensory cortex (BA3a, BA3), in the right superficial amygdala, entorhinal cortex extending into the piriform cortex, and bilaterally in the middle cingulate cortex (Figure 3/red, Table 3). The reverse contrast O^0^ > O^−^ showed significant BOLD responses in the right fusiform gyrus, the right hippocampus (EC and SUB), the right inferior temporal lobe and the left precuneus, with no gender differences.

**Table 3:** Activations during negative > neutral olfactory stimulation (O^−^ > O^0^).

				**MNI coordinates**		
**Area**	**Hemisphere**	**Cluster**	**Cluster extend**	**x**	**y**	**z**	***t*-value**
Anterior insula	R	C1	2,467	40	4	−12	10.69
Parietal operculum (OP4, OP1)	R	C1		54	−10	14	9.81
Rolandic operculum (BA44)	R	C1		62	6	4	7.72
Temporal pole	R	C1		50	14	−12	6.74
Precentral gyrus (BA6)	R	C1		62	2	26	6.60
Inferior parietal cortex (PFop)	R	C1		without peak voxel in the cluster			
Parietal operculum (OP4, OP1)	L	C2	1,131	−56	−4	12	8.31
Precentral gyrus (BA6)	L	C2		−62	2	28	7.46
Postcentral gyrus (BA1)	L	C2		−60	−4	38	6.23
Inferior parietal cortex (PFop, PFt)	L	C2		without peak voxel in the cluster			
Primary somatosensory cortex (BA3a, 3b)	L	C2		without peak voxel in the cluster			
Anterior insula	L	C3	948	−34	10	8	8.63
Temporal pole	L	C3		−52	16	−10	5.28
Cerebellum	L	C4	718			
Lobule VI				−26	−66	−26	7.62
Lobule VIIa Crus I				−38	−52	−36	7.22
Cerebellum (lobule VI)	R	C5	488	6	−74	−14	6.58
Postcentral g yrus (BA4p)	L	C6	146			
Precentral gyrus (BA4a)	R	C7	118	42	−10	38	6.41
Primary somatosensory cortex (BA3a, 3b)	R	C7		38	−14	34	5.97
Amygdala (SF, EC)	R	C8	113	20	0	−18	7.44
piriform cortex	R	C8		without peak voxel in the cluster			
Middle cingulate cortex	L	C10	17	−4	14	42	5.37
Middle cingulate cortex	R	C11	11	6	20	30	5.11

*FWE-corrected at p < 0.05 with extend threshold of 5 voxels. Presentation of areas with at least 5 % activation and peak voxel if possible*.

#### Interaction of Auditory and Olfactory Stimulation

**Figure 4:**
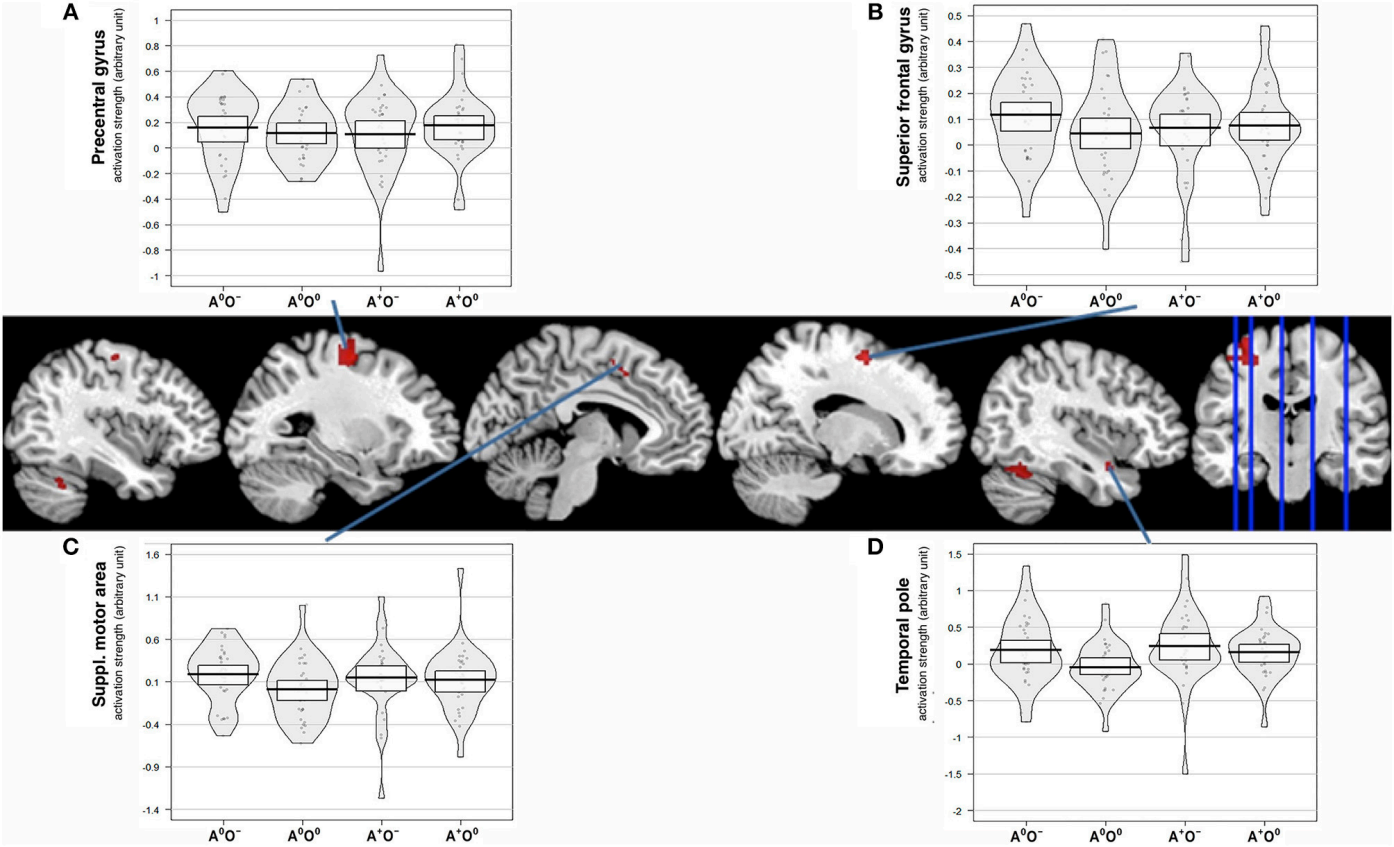
Interaction effect of auditory and olfactory stimulation. Activation strength and BOLD responses for conjunction analysis of interaction and main effect of positive auditory during neutral olfactory stimulation, (A × O) ∩ (A + O^0^) and negative olfactory stimulation during neutral auditory stimulation (A × O) ∩ (A^0^O^−^) as well as the three-way conjunction (A × O) ∩ (A + O^0^) ∩ (A^0^O^−^), uncorr. *p* < 0.001. **(A)** displays activation strength plots of mean values extracted from the precentral gyrus (peak voxel MNI:−26/-12/56), **(B)** displays activation strength plots of mean values extracted from the superior frontal gyrus (peak voxel MNI: 16/0/56) **(C)** displays activation strength plots of mean values extracted from the supplementary motor area (peak voxel MNI:−8/8/44), and **(D)** displays activation strength plots of mean values extracted from the temporal pole (peak voxel MNI: 40/4/-22).

Of the two directed interactions (A: A × O) n (A^+^O^0^) n (A^0^O^−^) and B: (A × O) n (A^+^O^−^) only the interaction that reflects attenuation of brain activation under negative olfactory and positive auditory stimulation yielded significant results. Significant BOLD responses were found in the left precentral gyrus (BA 6) including the precentral and superior frontal sulci, bilaterally in lobule VI and VIIa of the cerebellum, in the right superior frontal gyrus, in the right temporal pole, and in the left SMA (Figure 4, Table 4).

**Table 4:** Conjunction analysis of interaction and main effect of positive auditory during neutral olfactory stimulation (A × O ∩ A^+^O^0^).

				**MNI coordinates**		
**Area**	**Hemisphere**	**Cluster**	**Cluster extend**	**x**	**y**	**z**	***t*-value**
Precentral gyrus (BA6)	L	C1	396	−26	−12	56	5.34
Cerebellum (Lobule VI)	R	C2	143	38	−58	−26	4.10
Cerebellum (Lobule VIIa)	R			40	−62	−26	3.98
Cerebellum (Lobule VIIa)	L	C3	52	−38	−54	−36	3.76
Cerebellum (Lobule VI)	L			−34	−60	−28	3.61
Superior frontal gyrus (BA6) / sensorimotor cortex	R	C4	51	16	0	56	4.74
Temporal pole	R	C5	29	40	4	−22	4.29
Supplementary motor area (SMA)	L	C6	24	−8	8	44	3.79

*Uncorrected at p < 0.001 for individual contrasts with extend threshold of 10 voxels; conjunction threshold at p < 0.000001. Presentation of peak voxel coordinates*.

#### Integration of Brain Activation and Behavioral Ratings

We aimed to associate the subjective ratings of disgust, music appreciation and subjective affective state with brain activation. For this purpose, we correlated the mean subject-wise rating scores with mean brain activation estimates from the significant interaction clusters. Listed are significant correlations (*p* < 0.05), correlations surviving false discovery rate correction are marked with an asterisk. For the left precentral gyrus, we found significant correlations only in A^+^O^−^ for the disgust rating (*r* = 0.443; *p* = 0.011), the emotional state rating (*r* = −0.439; *p* = 0.012) and A^0^O^−^ for the disgust rating (*r* = 0.437; *p* = 0.012). In the left SMA, we found significant correlations in A^+^O^−^ for the disgust rating (*r* = 0.509; *p* = 0.003) and the emotional state rating (*r* = −0.362; *p* = 0.043) and in A^0^O^−^ for the disgust rating (*r* = 0.435; *p* = 0.01). In the superior frontal gyrus, we only found a significant correlation in A^+^O^−^ with the emotional state rating (*r* = −0.384; *p* = 0.03). The temporal pole showed significant correlations in A^+^O^−^ with the disgust rating (*r* = 0.624; *p* < 0.0001^*^), the music rating (*r* = −0.352; *p* = 0.048), and the rating of emotional state (*r* = −0.563; *p* < 0.001^*^). In A^0^O^−^ we observed a significant correlation with the disgust rating (*r* = 0.627; *p* < 0.001^*^). Correlations between rating scores and brain activity are reported in Supplementary Figures 1–4.

## Discussion

In the present study we were able to demonstrate that affective rivalry of auditory and olfactory stimuli shows complex behavioral and neuronal interaction effects. While the perceived level of disgust in reaction to the odor is solely modulated by the olfactory stimulation, the rating of the music is modulated by both the auditory condition and the olfactory condition. The affective state is modulated by both olfaction and audition, yet the interaction term indicates a stronger influence of olfaction on the subjective affective state.

This pattern implicates that automatic emotion regulation seems to be largely working uni-directionally in the case of negative olfaction paired with positive music. The music is not relieving the negative affect caused by the smell, but the smell is tainting the enjoyment of the music and the overall affective state. From an evolutionary perspective this is highly adaptive, because aversive odors are important to alert us when a potentially dangerous situation emerges and should not be easily influenced by other stimuli (e.g., positive auditory stimulation).

BOLD responses during negative, compared to neutral, olfactory stimulation include brain areas typically associated with processing olfactory signals and disgust, such as anterior parts of the insula and the piriform cortex (Zelano et al., 2005; Kurth et al., 2010; Reske et al., 2010; Lundström et al., 2011; Seubert et al., 2013a,b). The pre- and post-central activations in primary somatosensory and motor areas may reflect facial expressions resulting from the disgusting stimulation or sniffing movements that might have occurred, although participants were instructed to breathe orally. Activations in the entorhinal cortex and especially in the superficial amygdala are associated with odor, especially with emotionally negative valenced stimulation (Amunts et al., 2005; Bzdok et al., 2013; Seubert et al., 2013a). Cerebellar brain activity has been associated with emotional tasks, but it has also been determined to play an important role in auditory processing (Petacchi et al., 2005; Habel et al., 2007a; Baumann and Mattingley, 2012; Stoodley et al., 2012).

Similarly, brain activity related to positive music are concordant with findings of former studies and include brain areas that are involved in processing auditory signals and emotions (Olson et al., 2007; Koelsch, 2014). The posterior thalamus and the medial colliculus are relay centers forwarding signals from the inner ear to the primary auditory cortices, of which TE1.0, TE1.1, and TE1.2 are known sub-regions (Morosan et al., 2001). Also TE3.0 is associated with auditory and linguistic tasks (Morosan et al., 2005), whereas the right temporal pole seems to play a role in auditory and emotional processing (Olson et al., 2007). The posterior insula has previously been shown to be involved in sensory and integrative aspects of processing auditory signals, but also of chemosensory stimuli (Kurth et al., 2010), similar to parts of the inferior parietal lobe additionally associated with attention and mental imagery (Petacchi et al., 2005; Baumann and Mattingley, 2012; Stoodley et al., 2012).

In conclusion, the main effects of positive compared to neutral music, and negative compared to neutral odor, show BOLD responses in areas typically associated with processing of music, emotion and olfactory signals, which is widely concordant with results of former studies.

Overall, the interaction patterns show overlap with areas involved in multisensory processing (Calvert et al., 2000; Macaluso et al., 2000; Driver and Noesselt, 2008), which has also been shown for olfaction (Osterbauer et al., 2005; Seo and Hummel, 2017). Interestingly, a study on multisensory integration and touch perception has found similar brain activity patterns which was interpreted as resulting from moderator influences of disgust evaluation (Croy et al., 2016).

The largest cluster in the precentral gyrus may relate to somatosensory associated activation. Activation in this area may reflect somatotopy. This may be done by controlling unconscious behavioral tendencies (e.g., facial expressions of disgust), as this area, in conjunction with the SMA, was activated in a study on strategic control over interfering stimuli showed activity in this area (Wolbers et al., 2006). In line with this interpretation, adjacent SMA activation supports unimodal processing as we observe attenuated activity in A^0^O^0^ and A^+^O^−^, which is supported by a previous study showing involvement of the SMA in unimodal visual and auditory stimulation, as opposed to bimodal (Johnson and Zatorre, 2005). This area may thus passively mediate modulation of stimuli from different modalities. SMA and the region identified as superior frontal gyrus are also involved in emotion regulation (Ray and Zald, 2012); both may support integration or modulation of stimuli from different modalities and therefore play an important role in the context of affective regulatory processes taking place in our design. In our study, elevated SMA activity, when paired with negative olfaction with positive audition, may reflect stronger multisensory processing which in turn triggers generation of an emotion or affective state.

We have previously suggested the importance of this particular role in the process of emotion regulation for the SMA (Kohn et al., 2014), specifically for automatic emotion regulation (Kohn et al., 2011; Pawliczek et al., 2013). Besides SMA, superior frontal and sensory cortices may also play an important role in automatic emotion regulation (McRae et al., 2008; Kohn et al., 2011). SMA and neighboring regions may mediate an automatic down-regulation of positive affect elicited by music. Aversive odor may trigger an automatic (embodied) emotional reaction mediated by the SMA, which counteracts the emotional reaction elicited by the auditory stimulus (Niedenthal, 2007; Kohn et al., 2011, 2014). This also aligns with interactions of attention processes and multisensory processing, during which both bottom-up and top-down influences have been found (Talsma et al., 2007, 2010; Talsma, 2015; Hartcher-O'Brien et al., 2017). The focus of attention has been shown to modulate the affective response to a stimulus (Rolls et al., 2008). Similarly, more implicit automatic sensory processes supported by the SMA may underlie integration of positive music and negative olfaction.

The temporal pole seems to selectively integrate and process information from the modalities relevant to the evaluative process and ultimately for selection of the adequate action. Given its strong structural connections with the prefrontal cortex and the amygdala (Olson et al., 2007) it may be anatomically very well-suited to serve such a purpose. Brain imaging studies also indicated that this brain area may be strongly involved in the evaluative integration of olfactory stimulation.

For all four brain regions, we observed at least one significant correlation of brain activation to subjective ratings. After correcting for multiple comparisons, only the temporal pole retained significance (all four show uncorrected correlations). Given the observed patterns, one can draw the conclusion that brain activation from the interaction is most strongly associated to behavioral ratings of disgust and emotional state. Additionally, this coupling is nearly exclusively observable and strongest during the combination of positive music and negative smell. This supports our notion of these areas reflecting mainly multisensory processing, and additionally may indicate that modulation of brain activation in these areas also guides conscious labeling of emotional state and valence of external stimuli. Thus, these areas may link integratory brain activation to emotional experience which is in line with the supposed association of these multisensory processing areas with automatic emotion regulation when having to integrate conflicting sensory information.

As a side note, we found an interesting interaction effect of gender, in which disgust ratings in men are lowered by positive auditory stimuli. Conversely, women seem to show an additive effect, in which the combination of negative odor and positive auditory stimuli leads to an elevated disgust rating. Two different effects seem to occur in men and women. Men seem to be more strongly biased by positive auditory stimuli in their ratings of disgust and display a “soothing” effect of positive audition, while women show an aggravation of negative emotion when positive auditory stimuli are combined with negative odors. The experience derived from the nose does not seem to be unbiased by auditory stimulation but shows a differential gender effect. To the knowledge of the authors no study on olfaction-audition interaction has found such an effect. There is evidence that men and women differ strongly in perception and regulation of emotional responses. For example, behavioral studies suggest that women perform better in emotion identification tasks, for example in decoding non-verbal emotional cues (McClure, 2000; Gur et al., 2010) and show higher affective arousal or expression of emotion in interactions (Brody and Hall, 2000). We have previously argued (Kohn et al., 2011) that women might focus more strongly on valence of stimuli and utilize brain regions underlying generation and processing of affect. Men, however, tend to automatically regulate their emotions (McRae et al., 2008) and involve brain regions linked to emotion regulation to that end. In this context, men may use the positive auditory stimuli as a means of automatic, affective counter-regulation, leading to an overall influence of auditory stimuli on ratings of disgust. Women on the other side may focus on affectivity regardless of valence, which is higher for the two valenced stimuli, thus leading to stronger affective arousal, which they may in turn attribute to the disgusting smell.

### Limitations

Subjective unpleasantness of an odor has been shown to be correlated with activation in the medial olfactory cortex, including the piriform and anterior entorhinal cortex, the anterior cingulate cortex and the mid OFC (Rolls et al., 2003; Grabenhorst et al., 2007). Although, the OFC is known as a key structure implicated in both olfaction and emotion, we did not observe activation in this particular region. Specifically, when pleasantness of odors has to be rated, the OFC seems to be involved (Rolls et al., 2008). Although we observe activation in piriform cortex, we cannot rule out the possibility that medial parts of the OFC were sub-optimally sampled by our protocol. Another possibility is that during the perception phase, subjects were not asked to rate olfaction or music, and the rating phase was explicitly modeled, which may have captured some variance in OFC activity. Additionally, the mere requirement to rate the stimuli on affectivity might have altered the percept. In effect this would potentially render our paradigm somewhat biased toward higher order cognition.

We did not assess subjective ratings of unpaired auditory and olfactory stimuli, and therefore we cannot be sure on valence of these stimuli. The factorial design was not balanced due to an omission of a positive odor and a negative auditory stimulus, as well as an imbalance of modalities (16 auditory and 1 olfactory stimulus). The addition of further conditions in the design would have rendered the experiment too long (over 50 min in the MR scanner), which might in several ways threaten the validity of the results, by for example inducing fatigue, decreasing motivation, elevated motion ad drift artifacts. Nevertheless, we would argue that the pairing with neutral stimulation can serve as a reasonable control condition and especially for positive music (paired with non-scented air) the values reflect unbiased reports. A procedure to avoid habituation to the negative olfactory stimulus was implemented, however, adaptation processes might still have occurred. Furthermore, we cannot rule out the possibility that the differences rather reflect a valence effect where negative stimulation wins over positive stimulation. We cannot refute this possible effect on the basis of our data, which would pose an interesting question for future research.

## Conclusion

In our study we were able to demonstrate that positive auditory and negative olfactory stimuli, when paired, lead to a predominance of olfaction on the rating of disgust, on the perceived valence of the music and on subjective affective state, which we interpret as an evolutionary sensible effect of protective nature. On the neuronal level, positive auditory and negative olfactory stimulation leads to brain activation patterns consistent with previous literature, while pairing both stimuli is associated with complex interactions in brain areas.

Pairing both stimuli was associated with multisensory integration related brain activity in a set of brain areas (supplementary motor area, temporal pole, superior frontal gyrus). Essentially, we argue that these integratory areas pave the way for automatic emotion regulation processes which may contribute to the diminished appreciation of music and altered emotional state when being subjected to negative smells and positive music.

## Author Contributions

MB-L Implementation of the study, Statistics, Imaging process, Programming, Writing, and Manuscript editing; UH Supervision, Infrastructure, Conceptualization, and Manuscript editing; A-KB Infrastructure, Writing, and Manuscript editing; JF Infrastructure, Supervision olfaction, and Manuscript editing; KL Conceptualization, Music evaluation, Supervision music, and Manuscript editing; FS Supervision, Infrasctructure, and Manuscript editing; KA Supervision, Infrastructure, and Manuscript editing; NK Implementation of the study, Statistics, Imaging process, Programming, Writing, and Manuscript editing.

### Conflict of Interest Statement

The authors declare that the research was conducted in the absence of any commercial or financial relationships that could be construed as a potential conflict of interest.
