# Supplementary material for: Implicit Affective Rivalry: A Behavioral and fMRI Study Combining Olfactory and Auditory Stimulation
Source: Front Behav Neurosci. 2018 Dec 18;12:313. doi: 10.3389/fnbeh.2018.00313 (PMC6305346; doi:10.3389/fnbeh.2018.00313)
Supplement: Supplementary file 1 [file Data_Sheet_1.PDF]

Supplementary material

Contents

Main effects versus low-level baseline 2
Supplementary Table 1: 2
Supplementary Table 2: 3
Supplementary Table 3: 3
Supplementary Table 4: 4
Scatterplots for correlations between rating scores and brain activity 6

Main effects versus low-level baseline

We have calculated low level baseline contrasts for all four condition and report the FWE corrected ($p < 0.05$, voxel-level) results in tables below.

Supplementary Table 1: Activations during negative OS and neutral AS trials (against low level baseline). Coordinates are reported in the MNI system. The results are ordered by cluster with all locations of local maxima listed.

Brain Area (for clusters the cluster number is listed)	Peak voxel t-value	MNI-coordinates			Hemisphere
**Cluster 1: 22405 voxels**					
**Middle Occipital Gyrus**	T = 20.94	-28	-82	20	L
**Lingual Gyrus**	T = 19.33	-12	-88	-6	L
**Lingual Gyrus**	T = 18.85	8	-84	-8	R
**Calcarine Gyrus**	T = 18.41	6	-92	-2	R+L
**Calcarine Gyrus**	T = 18.36	8	-90	0	R
**V2**	T = 17.57	8	-96	10	R
**Fusiform Gyrus**	T = 17.41	26	-82	-10	R
**Middle Occipital Gyrus**	T = 16.91	-22	-94	20	L
**Superior Occipital Gyrus**	T = 16.78	16	-96	18	R
**Inferior Occipital Gyrus**	T = 15.29	-30	-78	-10	L
**Middle Occipital Gyrus**	T = 15.04	28	-90	14	R
**Cluster 2: 823 voxels**					
**Middle Frontal Gyrus**	T = 10.86	26	-2	50	R
**Superior Frontal Gyrus**	T = 6.32	34	2	64	R
**Cluster3: 499 voxels**					
**Posterior-Medial Frontal**	T = 8.35	-6	14	46	L
**Posterior-Medial Frontal**	T = 7.42	-8	6	56	L
**Posterior-Medial Frontal**	T = 7.35	-6	8	54	L
**Cluster4: 145 voxels**					
**Precentral Gyrus**	T = 8.35	46	2	30	R
**Cluster5: 116 voxels**					
**Inferior Frontal Gyrus (pars triangularis)**	T = 6.35	-52	26	28	L
**Inferior Frontal Gyrus (pars triangularis)**	T = 6.26	-46	26	26	L
**Cluster6: 78 voxels**					
**Middle Frontal Gyrus**	T = 5.81	42	40	32	R
**Cluster7: 24 voxels**					
**Middle Frontal Gyrus**	T = 5.64	48	32	34	R
**Middle Temporal Gyrus**	T = 5.37	-50	-38	0	L
**Middle Frontal Gyrus**	T = 8.35	46	2	30	R

Supplementary Table 2: Activations during neutral OS and neutral AS trials (against low level baseline). Coordinates are reported in the MNI system. The results are ordered by cluster with all locations of local maxima listed.

Brain Area (for clusters the cluster number is listed)	Peak voxel t-value	MNI-coordinates			Hemisphere
**Cluster 1: 20412 voxels**					
**Middle Occipital Gyrus**	T = 22.45	-28	-82	20	L
**Lingual Gyrus**	T = 18.77	-12	-88	-6	L
**Lingual Gyrus**	T = 17.91	10	-84	-10	R
**Fusiform Gyrus**	T = 17.85	26	-82	-10	R
**V2**	T = 17.53	8	-96	10	R
**Superior Occipital Gyrus**	T = 17.51	16	-96	18	R
**Middle Occipital Gyrus**	T = 17.31	30	-90	14	R
**Calcarine Gyrus**	T = 17.15	6	-92	0	L
**Superior Parietal Lobule**	T = 15.27	-24	-62	50	L
**Inferior Occipital Gyrus**	T = 14.72	-30	-78	-10	L
**Inferior Occipital Gyrus**	T = 13.29	-44	-78	-10	L
**Cluster2: 456 voxels**					
**Superior Frontal Gyrus**	T = 10.58	26	-2	52	R
**Cluster3 207 voxels**					
**Precentral Gyrus**	T = 8.21	-42	0	32	L
**Cluster4: 85 voxels**					
**Posterior-Medial Frontal**	T = 5.77	-6	12	48	L
**Posterior-Medial Frontal**	T = 5.70	-8	8	54	L
**Cluster 5: 84 voxels:**					
**Precentral Gyrus**	T = 8.24	44	2	30	R

Supplementary Table 3: Activations during negative OS and positive AS trials (against low level baseline). Coordinates are reported in the MNI system. The results are ordered by cluster with all locations of local maxima listed.

Brain Area (for clusters the cluster number is listed)	Peak voxel t-value	MNI-coordinates			Hemisphere
**Cluster 1: 21769 voxels**					
**Middle Occipital Gyrus**	T = 21.20	-30	-80	20	L
**Lingual Gyrus**	T = 18.95	8	-84	-8	R
**Lingual Gyrus**	T = 18.67	-12	-88	-8	L
**Calcarine Gyrus**	T = 18.47	8	-90	0	R
**Calcarine Gyrus**	T = 18.41	6	-92	-2	R
**Fusiform Gyrus**	T = 17.80	26	-82	-10	R
**V2**	T = 17.63	8	-96	10	R
**Superior Occipital Gyrus**	T = 16.77	16	-96	18	R

**Middle Occipital Gyrus**	T = 16.64	-22	-94	20	L
**Middle Occipital Gyrus**	T = 15.43	28	-90	14	R
**Inferior Occipital Gyrus**	T = 14.91	-30	-78	-10	L
**Cluster2: 531 voxels**					
**Temporal Pole**	T = 8.88	58	4	-8	R
**Cluster 3: 476 voxels**					
**Superior Frontal Gyrus**	T = 9.73	26	-2	52	R
**Superior Frontal Gyrus**	T = 5.82	34	2	64	R
**Cluster4: 394 voxels**					
**Posterior-Medial Frontal**	T = 7.53	-8	6	58	L
**Posterior-Medial Frontal**	T = 7.49	-6	14	44	L
**Cluster5: 354 Voxels**					
**Superior Temporal Gyrus**	T = 6.97	-58	-4	0	L
**Superior Temporal Gyrus**	T = 6.31	-60	10	-6	L
**Superior Temporal Gyrus**	T = 5.15	-68	-14	2	L
**Cluster 6: 59 voxels**					
**Precentral Gyrus**	T = 7.05	44	2	30	R
**Cluster 7: 35 voxels**					
**Inferior Parietal Lobule**	T = 5.52	50	-40	50	R
**Cluster 8: 25 voxels**					
**Middle Frontal Gyrus**	T = 5.31	42	40	32	R
**Middle Frontal Gyrus**	T = 5.20	46	34	34	R
**Cluster 9: 24 voxels**					
**Inferior Frontal Gyrus (pars Triangularis)**	T = 5.39	-54	26	28	L
**Inferior Frontal Gyrus (pars Triangularis)**	T = 5.08	-46	26	26	L
**Cluster 10: 19 voxels:**					
**Temporal Pole**	T = 5.34	40	4	-20	R

Supplementary Table 4: Activations during neutral OS and positive AS trials (against low level baseline). Coordinates are reported in the MNI system. The results are ordered by cluster with all locations of local maxima listed.

Brain Area (for clusters the cluster number is listed)	Peak voxel t-value	MNI-coordinates			Hemisphere
**Cluster 1: 22923 voxels**					
**Middle Occipital Gyrus**	T = 21.20	-30	-80	20	L
**Lingual Gyrus**	T = 23.32	-30	-80	20	L
**Fusiform Gyrus**	T = 20.42	-12	-88	-6	L

**Lingual Gyrus**	T = 20.23	26	-82	-10	R
**Calcarine Gyrus**	T = 19.82	8	-84	-8	L+R
**V2**	T = 18.88	6	-92	-2	R
**Superior Occipital Gyrus**	T = 18.82	8	-96	10	R
**Middle Occipital Gyrus**	T = 18.39	16	-96	18	R
**Inferior Occipital Gyrus**	T = 18.19	30	-90	14	R
**Superior Parietal Lobule**	T = 16.43	-30	-80	-10	L
**Inferior Occipital Gyrus**	T = 15.97	-20	-64	52	L
**Middle Occipital Gyrus**	T = 15.49	-44	-78	-10	L
**Cluster2: 556**					
**Precentral Gyrus**	T = 10.87	26	-4	50	R
**Cluster 3: 405 voxels**					
**Superior Temporal Gyrus**	T = 9.69	56	0	-8	R
**Cluster 4: 215 voxels**					
**Superior Temporal Gyrus**	T = 7.16	-56	-4	-2	L
**Superior Temporal Gyrus**	T = 5.65	-60	8	-8	L
**Cluster5: 159 voxels**					
**Posterior-Medial Frontal**	T = 6.71	-6	12	48	L
**Cluster6: 36 voxels**					
**Precentral Gyrus**	T = 6.78	44	2	30	R
**Cluster 7: 23 voxels**					
**Superior Temporal Gyrus**	T = 5.61	-68	-18	4	L
**Superior Temporal Gyrus**	T = 5.42	-68	-14	2	L
**Cluster 8: 8 voxels**					
**Thalamus**	T = 5.39	-24	-26	-2	L

Scatterplots for correlations between rating scores and brain activity

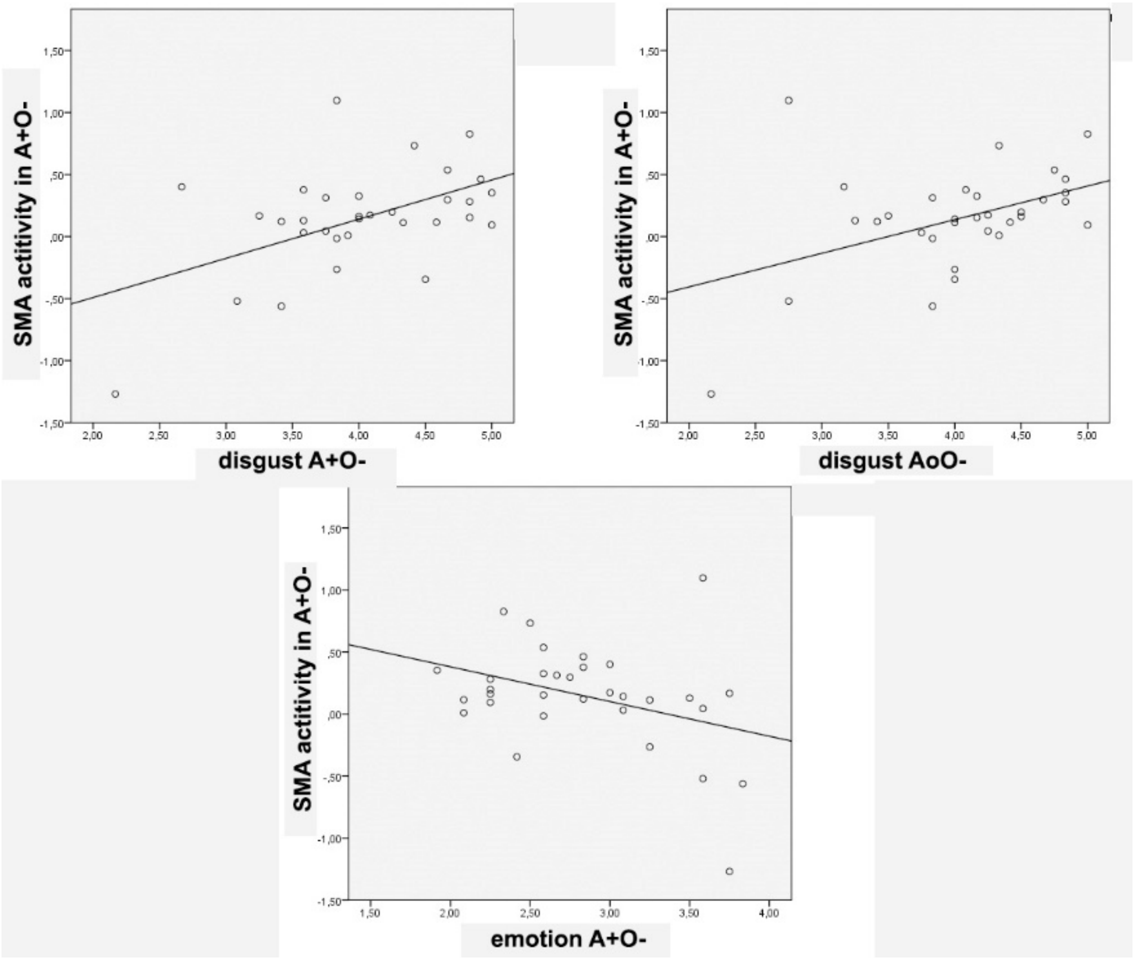

Figure 1: SMA activity was correlated with three rating scores: in A^+O^- for the disgust rating (r = 0.509; p = 0.003) and the emotional state rating (r = -0.362; p = 0.043) and in A^0O^- for the disgust rating (r = 0.435; p = 0.01).

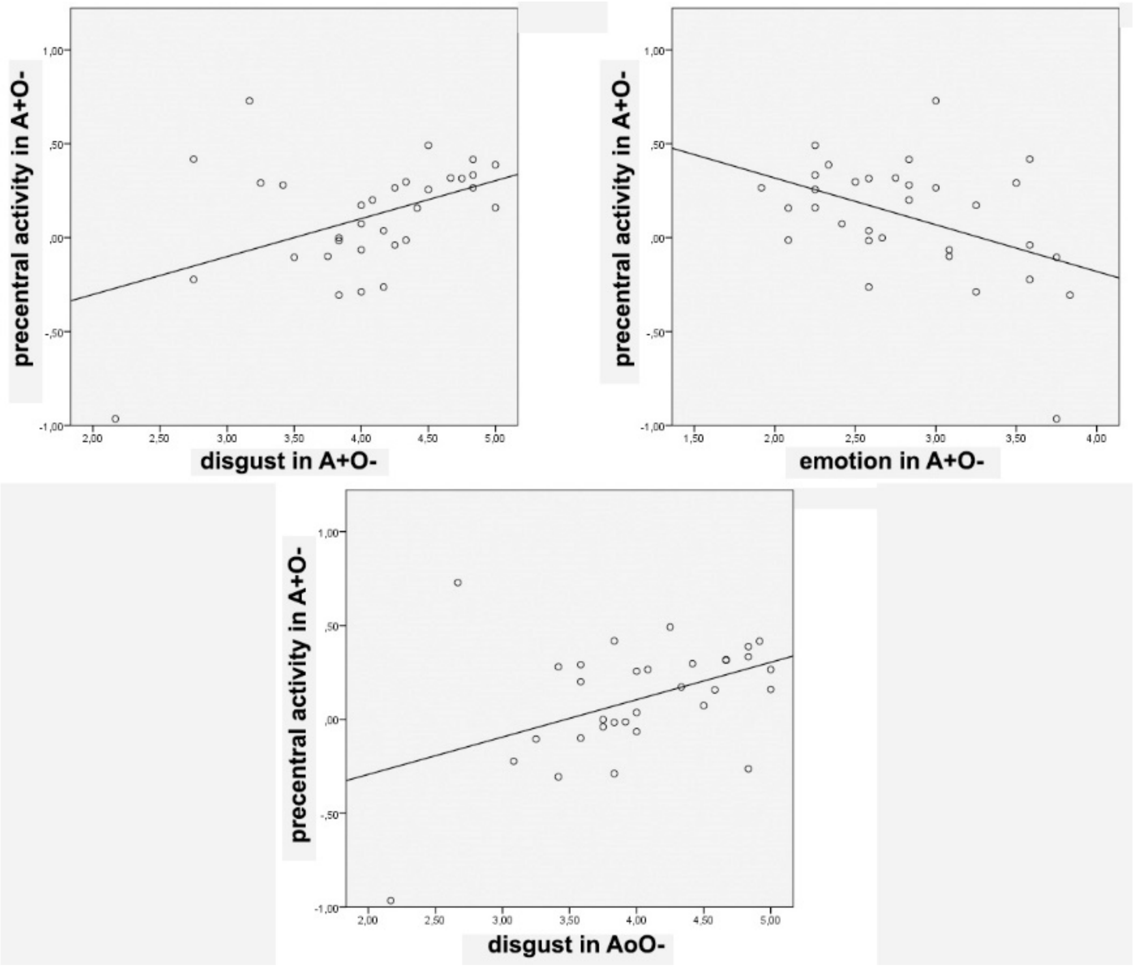

Figure 2: precentral activity was also correlated with three ratings scores: in A^+O^- for the disgust rating ($r = 0.443$; $p = 0.011$), the emotional state rating ($r = -0.439$; $p = 0.012$) and A^0O^- for the disgust rating ($r = 0.437$; $p = 0.012$).

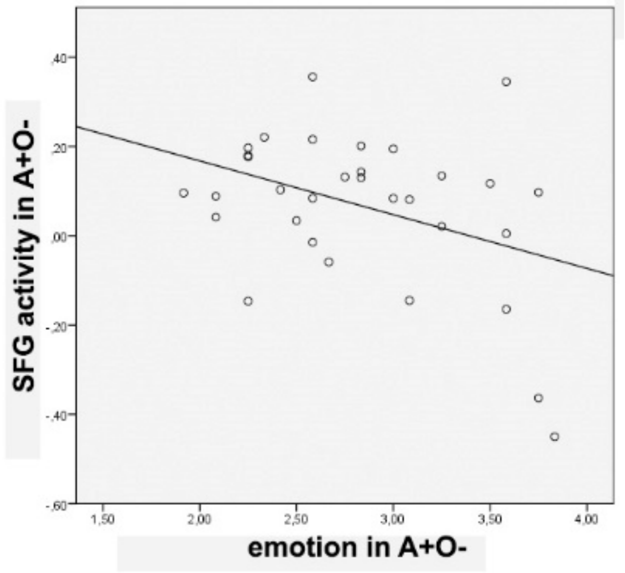

Figure 3: Activity in the SFG was correlated with the emotional state rating in A^+O^- ($r = -0.384$; $p = 0.03$)

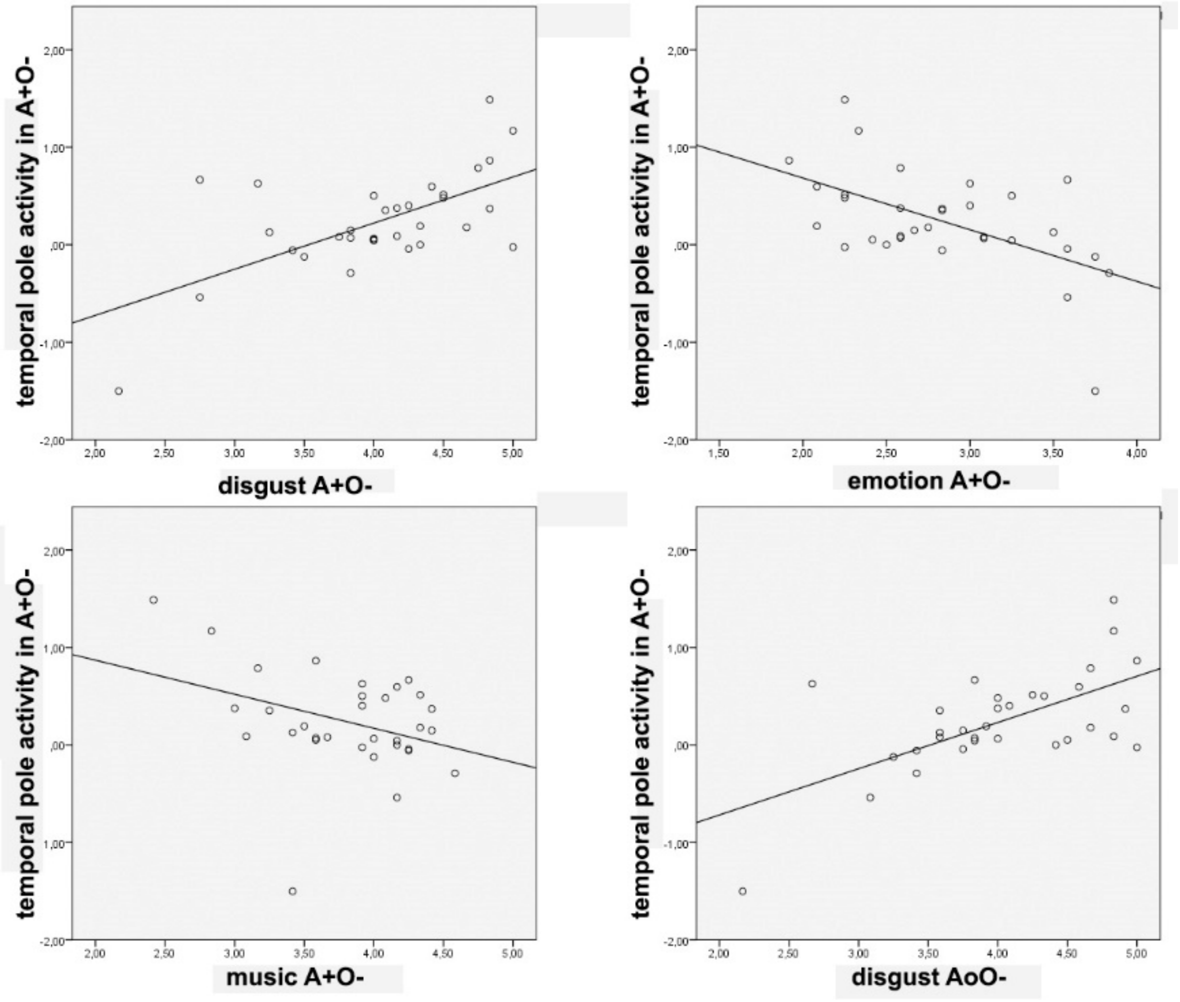

Figure 4: Temporal pole activity was correlated with four ratings: in A+O- with the disgust rating (r = 0.624; p < 0.0001), the music rating (r = 0.352; p = 0.048), and the rating of emotional state (r = 0.563; p < 0.001*). In A0O- we observed a significant correlation with the disgust rating (r = 0.627; p < 0.001*).*
